# HaTSPiL: A modular pipeline for high-throughput sequencing data analysis

**DOI:** 10.1371/journal.pone.0222512

**Published:** 2019-10-15

**Authors:** Edoardo Morandi, Matteo Cereda, Danny Incarnato, Caterina Parlato, Giulia Basile, Francesca Anselmi, Andrea Lauria, Lisa Marie Simon, Isabelle Laurence Polignano, Francesca Arruga, Silvia Deaglio, Elisa Tirtei, Franca Fagioli, Salvatore Oliviero

**Affiliations:** 1 Department of Life Sciences and System Biology, University of Turin, Turin, Italy; 2 Italian Institute for Genomic Medicine (IIGM), Turin, Italy; 3 Department of Medical Sciences, University of Turin, Turin, Italy; 4 Paediatric Onco-Haematology, Stem Cell Transplantation and Cellular Therapy Division, City of Science and Health of Turin, Regina Margherita Children’s Hospital, Turin, Italy; University of Helsinki, FINLAND

## Abstract

**Background:**

Next generation sequencing methods are widely adopted for a large amount of scientific purposes, from pure research to health-related studies. The decreasing costs per analysis led to big amounts of generated data and to the subsequent improvement of software for the respective analyses. As a consequence, many approaches have been developed to chain different software in order to obtain reliable and reproducible workflows. However, the large range of applications for NGS approaches entails the challenge to manage many different workflows without losing reliability.

**Methods:**

We here present a high-throughput sequencing pipeline (HaTSPiL), a Python-powered CLI tool designed to handle different approaches for data analysis with a high level of reliability. The software relies on the barcoding of filenames using a human readable naming convention that contains any information regarding the sample needed by the software to automatically choose different workflows and parameters. HaTSPiL is highly modular and customisable, allowing the users to extend its features for any specific need.

**Conclusions:**

HaTSPiL is licensed as Free Software under the MIT license and it is available at https://github.com/dodomorandi/hatspil.

## Introduction

Large-scale biological data analysis often involves many domain-specific tools chained together in order to obtain meaningful results. The handling of workflows is a well-known problem in computer science, and many software have been specifically developed. For instance, Galaxy [1], Taverna [2], GeneProf [3] and Sequanix [4] are user-friendly GUI-based interfaces aimed at creating custom pipelines for non-programmer users. At the same time, other solutions have been developed for more advanced users to provide more control over operations using existing general-purpose programming languages or domain-specific languages (DSL). Ruffus [5] pipelines are created using the Python language, Pwrake [6] and GXP Make [7] are based on the existing DSL Rake and Make. BigDataScript [8], Bpipe [9] and SnakeMake [10] are examples of languages specifically designed for the development of bioinformatics pipelines, aiming at the maximum flexibility and powerfulness without the steeper learning curve of a general-purpose programming language. It is worth noting that Sequanix is a GUI to SnakeMake, demonstrating that it is possible to obtain high accessibility without sacrificing flexibility and powerfulness. There are other systems that can be used to create reliable workflows, but they have slightly different aims or they are difficult to extend with new tools [11].

Nevertheless, all these approaches suffer some limitations. First, samples from different sources have to be analysed with similar approaches but with specific implementations. This problem could be solved by performing small sample-specific modifications to the pipeline to obtain the desired results. This approach is highly error-prone, as every change made to the pipeline might introduce new errors producing inconsistent results. Moreover, the possibility to multiplex many NGS experiments in a single sequencing run introduces the need to perform different analyses with different parameters on distinct files.

To tackle these issues, we here introduce HaTSPiL, a python-based pipeline for high-throughput sequencing data analyses designed with the idea of being modular, flexible and expandable. HaTSPiL automatises all steps of an NGS analysis through the use of a barcoding system encoding all the required information, thus minimising user’s intervention. HaTSPiL is based on two layers: a core layer responsible for the execution of all the parts of the workflow and an external layer that defines each step of the pipeline. To show the functionality of HaTSPiL, we set it up to perform mutation analysis on DNA sequencing data (i.e. whole exome or targeted gene sequencing). HaTSPiL can be easily modified by users to customise solutions and extend the capabilities of the software.

## Materials and methods

### Implementation

HaTSPiL is both a command line tool and a Python library. There are some core modules that are responsible for handling the complete workflow process, from the evaluation of the files that have to be processed, to the interconnection between all the steps of the pipeline. These core functionalities can be summarised in four major topics, each of them organised in different python modules: initialisation, configuration, barcoding and execution. A set of modules are written over these functionalities to assess the mutations of various kinds of samples and to generate the relative reports. Every major topic discussed below has a different level of customisability: basics Python programming skills are needed to introduce new features in some parts of HaTSPiL, others require a very good knowledge of the language and deep understanding of the whole software in order to correctly modify its behaviour. (See the *README* in the repository for a customisation example).

#### Initialisation

The initialisation step is handled by the *hatspil* module. This module evaluates the input parameters provided by the user, loads a valid configuration for the workflow, finds the required files and chooses how they need to be handled. The initialisation process can be subdivided into two phases. A first phase consists of multiple coherence checks, which dynamically evaluate the presence of eventual problems. The second phase decodes the information contained in the barcodes (see below) and, given case-control analyses, it matches cases with the relative controls. These two phases ensure both the reliability and reproducibility of the analysis workflows, even if they are run on different dates and with different versions of the software.

The main customisation points for this core part are related to the addition of command line parameters that can be applied to all the different samples that are analysed in a single run.

The *hatspil* module does not start every step of the workflow, but it delegates this responsibility to the *runner* module that executes the different parts of the pipeline. The *runner* module can be customised to expand HaTSPiL functionalites. Its physical separation from the *hatspil* module simplifies the process. Once a new functionality is added to HaTSPiL through the introduction of a new module, only a few lines of code need to be added inside the *runner* in order to extend the software.

#### Configuration

During the initialisation phase, HaTSPiL performs a configuration phase using a .*ini* file. This file contains all the information that are constant for every possible workflows (e.g. paths of the executables, default parameters as memory usage and other global preferences). This information is parsed by the *config* module that checks for the presence of all the required fields in the .*ini* file, their default values and performs preliminary tests on the third-party executable files.

New parameters can be added to the *config* module by placing them in the appropriate category list (files, executables, *jars* and so on) and specifying the default parameters. All the configuration data is pervasive across many parts of the software thanks to the *analysis* module (see Execution below), therefore it is easy to use this information in the workflow modules.

#### Barcoding

One of the major advantages of HaTSPiL consists in the use of a highly reliable barcoding system. This approach, similar to the one used by The Cancer Genome Atlas project [12], is particularly convenient in presence of high-volume sequencing experiments.

The barcode consists of a set of parameters which allow a unique identification of the respective samples (i.e. project, tissue, biopsy, whether it is a xenograft, etc.) and, at the same time, it provides information aimed at automatically executing all the required analysis steps. The barcode structure allows to work with different type of samples and distinct experimental settings (e.g. DNA-seq, RNA-seq, etc.) enabling to track several types of analyses.

Barcode fields (see Text in S1 Text) are handled by the *barcoded_filename* module, which controls the consistency of information and allows a straightforward access to the characteristics of the sample. The handling of the barcode can be customised by the users, allowing the integration of new functionalities.

#### Execution

Each module of the workflow uses the same basic structures to perform different analyses. The first structure is the configuration of the workflow discussed above, and the second structure is defined in the *analysis* module. This module is responsible for the storage of the information between different steps of the workflow. Each step can handle one or more input data and one or more output files. Moreover, some steps can be optional and input files can change depending on the parameters and barcode fields. The output data of each step is stored by the *analysis* module to provide meaningful information for a possible subsequent phase.

The execution of every part of the workflow is performed by the *executor* module module. When a workflow module runs a step through the *executor*, the arguments of the external program, which can be specified using pseudo-variables, will be interpreted and replaced with the appropriate values depending on the state of the *analysis*. Furthermore, many customisable parameters can be used to explicitly change the behaviour of the *executor* module. This module handles all the possible combinations of parameters and *analysis* statuses, providing high flexibility to HaTSPiL.

## Results

**Fig 1:**
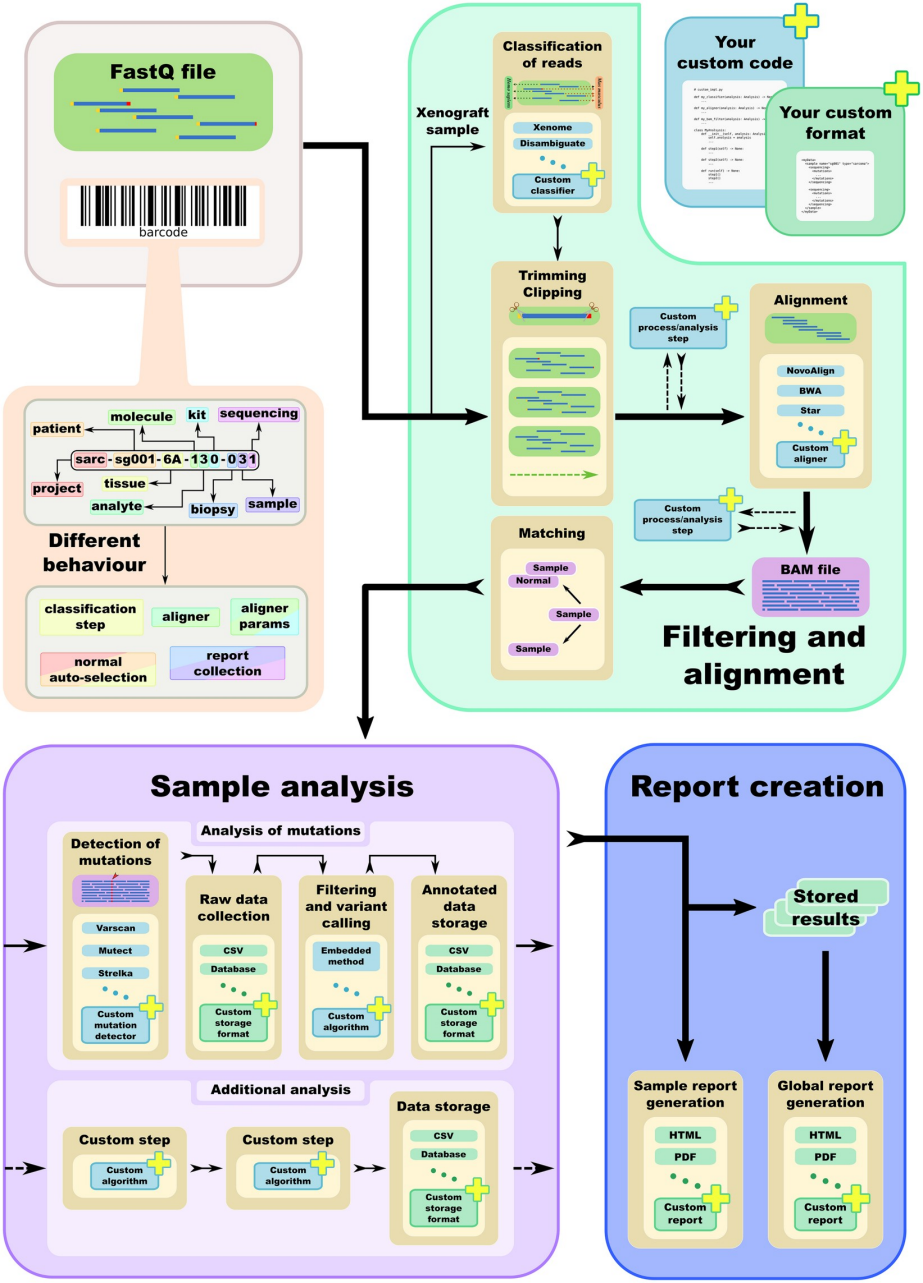
A schematic representation of the workflow of HaTSPiL. The topmost left part shows the starting point of the whole analysis, a set of barcoded FastQ files. The software supports the most common operations to handle the data, performing different steps of filtering and alignment. It is shown that the various steps can change depending on the barcoding of the sample, and that the workflow is highly customisable. At date, HaTSPiL supports a mutation analysis pipeline, and this feature will be improved and extended. Additional analysis pipeline can be added easily as well, and a final step of report generation is included, in order to provide an immediate and user-friendly output.

HaTSPiL was developed to solve practical problems related to NGS data analysis, being its usability and reliability the highest priority of the project. The tool comes with a bundled workflow for mutation analysis of DNA-sequencing experiments (both whole exome or whole genome sequencing) and it has been successfully run on different types of tumours, xenografts, blood derived samples and cell organoids. The software automatically matches the tumour samples with the relative control samples to improve the detection of somatic mutations. The workflow relies on the utilisation of widely used software in order to take advantage of the state-of-the-art solutions available to date. On the other hand, specific cases required the development of custom analysis scripts (Fig 1).

**Fig 2:**
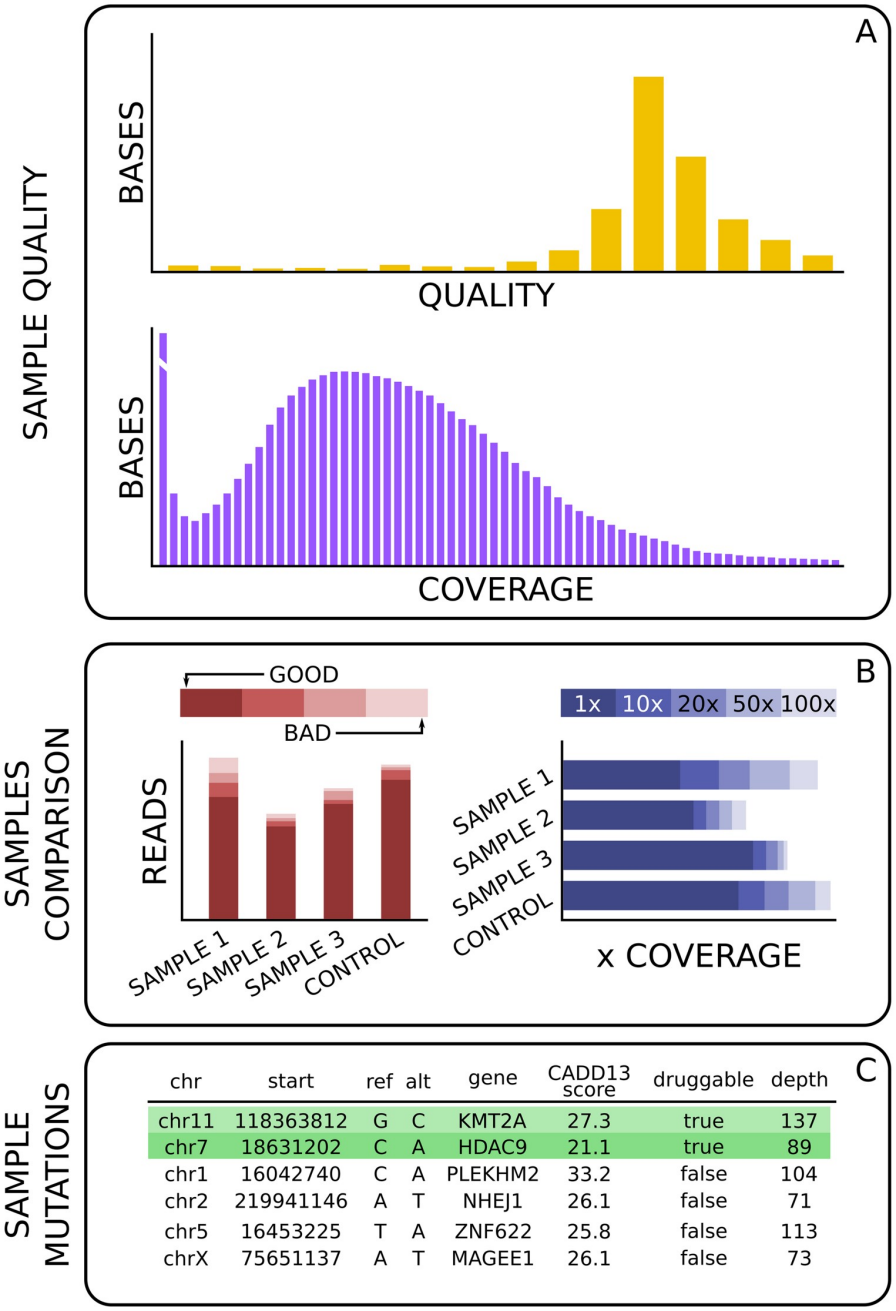
Representation of the output in a report automatically generated by HaTSPiL. (A) Two of the plots showing the quality of a single sequencing. The values are obtained using Picard software (https://broadinstitute.github.io/picard/). (B) Two comparisons between samples and relative control. This representation is used with a scalable amount of samples, from a single sample to all the samples available for a whole project. A control sample is always added, if available, in order to provide a minimal comparison against another sequencing. (C) A portion of the table containing the mutations found highly damaging by the variant calling. The mutated genes that are known to be a target of a drug are highlighted.

HaTSPiL can also automatically generate reports, thus providing a user-friendly interface to check results. For every set of input files, interactive plots are created to show the quality and the coverage of the samples (Fig 2A). Graphical comparisons between samples and relative controls are shown when available (Fig 2B). An interactive table with the damaging mutations found by the variant calling process is also shown, highlighting the genes that are known to be druggable (Fig 2C). HaTSPiL is also capable of reading all the data stored during previous analyses and to generate a summary for every sequencing, layering the information by sample, biopsy, patient and project. This simple representation provides the user with an overview of the experiments across a large timescale, facilitating the detection of problems and biases for specific sequencing experiments.

## Discussion

The high customisability and modularity of HaTSPiL allows the implementation of many steps that can be useful for the user, even if the aim is different from mutation analysis. Adapter trimming is performed through Cutadapt [13], which can be run for both single- and paired-end sequencing and which uses the parameters specified in the *config.ini* file. The FastQC tool (https://www.bioinformatics.babraham.ac.uk/projects/fastqc/) is run to produce a glimpse of the overall sequencing quality, then a quality trimming is performed through SeqKit [14] to avoid common quality loss at the start and the end of the reads. HaTPSiL then performs a sequence alignment using one of the supported software: NovoAlign (http://www.novocraft.com/products/novoalign/) or BWA [15] for DNA-based sequencing, or STAR [16] for RNA-seq analyses. It is also possible to handle xenograft samples, which require a step aimed at splitting the reads belonging to the tumour from those belonging to the avatar animal, using either Xenome [17] or Disambiguate [18]. The data obtained from the alignment step is then refined using GATK [19] and Picard (https://broadinstitute.github.io/picard/) in order to perform indel realignment, base recalibration, removal of duplicates and collection of statistics. All the described steps are common to many different NGS analysis pipelines, therefore HaTSPiL can be extremely useful in a large number of situations, even without any need for user customisation. Moreover, a mutation analysis procedure is automatically run using Mutect [20], VarScan [21] and Strelka [22], by automatically changing the behaviour of the workflow and the used parameters depending on the presence of control samples. The mutations found by these software are further evaluated and filtered using a previously published method, which allows the integration of clinical information from curated databases as Cosmic [23], dbSNP [24] and ClinVar [25].

## Conclusion

HaTSPiL is a powerful workflow engine for next-generation sequencing data analysis, aiming at higher reliability, modularity and reproducibility. The approach is orthogonal to other existing systems for the automation of chained bioinformatics operations, and the software is designed to be used in different fields, from pure research to health-related projects. The software is designed to be easily employed by both non-experienced (as a ready-to-use pipeline) and experienced users (due to its great customisability and extendibility). It is worth noting that the large API and the database interoperability allow for the creation of third-party software that can take advantage of the reliability of the engine. HaTSPiL will continuously improve, through the integration of many different NGS data analysis tools and workflows. In future releases we will expand HaTSPil through the integration of new features and better support for auxiliary software.

## Supporting information

S1 Text Barcode specification. A brief description of the barcode and its fields.
(PDF)

S2 Text Xenograft sample. Explanation of how xenograft sample information is integrated into the barcode.
(PDF)

S1 Table Comparison table. A brief comparison between pipeline software.
(PDF)
