# Supplementary material for: HaTSPiL: A modular pipeline for high-throughput sequencing data analysis
Source: PLoS One. 2019 Oct 15;14(10):e0222512. doi: 10.1371/journal.pone.0222512 (PMC6793853; doi:10.1371/journal.pone.0222512)
Supplement: S1 Text — A brief description of the barcode and its fields. (PDF) [file pone.0222512.s001.pdf]

Barcode specifications The barcode is composed by different fields, and each field has a different meaning and characteristics (see Fig 1).

- **project**: generally, samples are analyzed in the context of a big project. This field is designed to help researchers and users to put together a large group of samples.
- **patient**: when the research field is strictly related to human health, it is necessary to distinguish people using anonymous strings. When human samples are not involved, this field can be used to distinguish between experiments.
- **tissue**: this field contains the type of tissue the sample is taken from, and the possible values are nearly the same as specified by the TCGA internal notation for samples (`https://gdc.cancer.gov/resources-tcga-users/tcga-code-tables/sample-type-codes`). The only mismatching codes are related to the notation for xenograft samples, which differs from the original one for technical details (see Text in S2 Text).
- **molecule**: identifies whether the sample is DNA (0) or RNA (1).
- **analyte**: the sample can be processed to perform different experimental analyses. The software currently supports *whole exome* data (0), *gene panel* data (1) and *fusion panel* data (2). It is possible to specify an *RNA-seq* data (3) in order to obtain a *raw* count per gene. Future releases will introduce new features to obtain more valuable information from RNA-seq data.
- **kit**: it is possible to handle a sample with different library preparation kits in order to obtain different results. This can be particularly useful when using different *gene panels* and *whole exome* libraries. HaTSPiL automatically selects different sets of parameters from the configuration file based on the *kit* and the *analyte* barcode values.
- **biopsy**: this field identifies the index of the biopsy from the same patient. This can be really useful in real case situations, in which it is important to monitor a person along the time. It currently supports indices from 0 to 9.
- **sample**: sometimes a biopsy can be quite non-homogeneous, and it could be necessary to perform multiple analyses on the same biopsy. This field is designed to be used in these cases, and it currently supports numbers from 0 to 9. In case the sample is from a xenograft tissue, this field contains coded information of the animal involved in the xenotransplantation (see Text in S2 Text).
- **sequencing**: this last field is designed to perform multiple experimental analyses on the same exact sample, with the same protocols. This is useful for reproducibility purposes and to overcome problems due to library preparation and sequencing issues.
