# Supplementary material for: HaTSPiL: A modular pipeline for high-throughput sequencing data analysis
Source: PLoS One. 2019 Oct 15;14(10):e0222512. doi: 10.1371/journal.pone.0222512 (PMC6793853; doi:10.1371/journal.pone.0222512)
Supplement: S2 Text — Explanation of how xenograft sample information is integrated into the barcode. (PDF) [file pone.0222512.s002.pdf]

Xenograft sample When a sample is obtained from a xenograft tissue, many additional aspects must be taken into account. In particular, it is necessary to store the information about the generation of the avatar animal. In the barcoding of HaTSPiL, this data is coded in the tissue and sample fields in order to avoid the *pollution* of a further field that is only useful in these specific cases.

In details, when the first character of the tissue field is a “6”, then the sample can only come from xenograft tissue. Moreover, in the case the second character is uppercase the sample is from a primary xenograft tissue, otherwise from cell lines derived from xenograft tissues. On the other hand, the value of the sample codes for the information of the generation of the animal, the identification of the *parental host* and the identification of the animal itself inside its generation. Given that is the second character of the tissue field of the barcode and is the value in the sample field, the information for the xenograft is decoded as following:

$$generation = lowercase\,(t) - \text{‘}a\text{’}$$
$$parent = \frac{s}{3}$$
$$sibling = mod\,(s, 2)$$

The limitations of this coding is that there are at maximum 26 generations, with at maximum 3 avatar animals obtained from the same *parental host*. These limitations rely on the fact that handling avatar animals is costly, and that three xenotransplantations from the same host are generally enough. To date, HaTSPiL is designed to handle xenograft samples from murine avatars, but it is possible to extend this feature to different animal species.
