# Supplementary material for: HaTSPiL: A modular pipeline for high-throughput sequencing data analysis
Source: PLoS One. 2019 Oct 15;14(10):e0222512. doi: 10.1371/journal.pone.0222512 (PMC6793853; doi:10.1371/journal.pone.0222512)
Supplement: S1 Table — A brief comparison between pipeline software. (PDF) [file pone.0222512.s003.pdf]

Comparison table The following is a brief comparison between pipeline software.

Software	Filename automation	General purpose	Focus on Bioinformatics	Basic NGS tools	GUI	Language
HaTSPiL	Yes	No	Yes	Yes	No	Python
GNU Make	Basic	Yes	No	No	No	Make
SnakeMake	No	Yes	Yes	No	No	Make-like
Rake	Basic	Yes	No	No	No	Ruby
Luigi	No	Yes	No	No	Yes	Python
Galaxy	No	No	Yes	Yes	Yes	GUI/JSON
Taverna	No	No	Yes	Yes	Yes	GUI/XML

Table 1. A brief comparison between HaTSPiL and some of the major software used for handling workflows. This generic comparison shows how different tools have been developed for different aims. Although a perfect tool for all the possible needs does not exist, each software focuses on a set of features to satisfy specific needs.
